# Cellular Vacuoles Induced by *Mycoplasma pneumoniae* CARDS Toxin Originate from Rab9-Associated Compartments

**DOI:** 10.1371/journal.pone.0022877

**Published:** 2011-07-29

**Authors:** Coreen Johnson, T. R. Kannan, Joel B. Baseman

**Affiliations:** Department of Microbiology and Immunology, The University of Texas Health Science Center at San Antonio, San Antonio, Texas, United States of America; Charité-University Medicine Berlin, Germany

## Abstract

Recently, we identified an ADP-ribosylating and vacuolating cytotoxin in *Mycoplasma pneumoniae* designated Community Acquired Respiratory Distress Syndrome (CARDS) toxin. In this study we show that vacuoles induced by recombinant CARDS (rCARDS) toxin are acidic and derive from the endocytic pathway as determined by the uptake of neutral red and the fluid-phase marker, Lucifer yellow, respectively. Also, we demonstrate that the formation of rCARDS toxin-associated cytoplasmic vacuoles is inhibited by the vacuolar ATPase inhibitor, bafilomycin A1, and the ionophore, monensin. To examine the ontogeny of these vacuoles, we analyzed the distribution of endosomal and lysosomal membrane markers during vacuole formation and observed the enrichment of the late endosomal GTPase, Rab9, around rCARDS toxin-induced vacuoles. Immunogold-labeled Rab9 and overexpression of green fluorescent-tagged Rab9 further confirmed vacuolar association. The late endosomal- and lysosomal-associated membrane proteins, LAMP1 and LAMP2, also localized to the vacuolar membranes, while the late endosomal protein, Rab7, and early endosomal markers, Rab5 and EEA1, were excluded. HeLa cells expressing dominant-negative (DN) Rab9 exhibited markedly reduced vacuole formation in the presence of rCARDS toxin, in contrast to cells expressing DN-Rab7, highlighting the importance of Rab9 function in rCARDS toxin-induced vacuolation. Our findings reveal the unique Rab9-association with rCARDS toxin-induced vacuoles and its possible relationship to the characteristic histopathology that accompanies *M. pneumoniae* infection.

## Introduction

*Mycoplasma pneumoniae*, the etiologic agent of primary atypical pneumonia, is associated with a wide variety of respiratory infections ranging from tracheobronchitis and pharyngitis to extrapulmonary infections, including joint and central nervous system disorders [1]. It is believed to be responsible for 20–30% of all community-acquired pneumonia and is also linked to acute and chronic airway diseases, such as bronchial asthma and chronic obstructive pulmonary disease [2], [3], [4], [5]. *M. pneumoniae* is generally considered an extracellular pathogen that adheres to mucosal epithelial surfaces, although it has the capability to replicate and survive intracellularly for extended periods [6], [7]. Cytadherence of *M. pneumoniae* to host cells is a prerequisite for colonization and infection, which is primarily achieved through an assemblage of proteins localized at its tip organelle [8], [9], [10]. However, alternate mechanisms of adherence to host cells have been reported by us and are likely to be physiologically relevant [11], [12]. Following mycoplasma infection, the cytopathology that *M. pneumoniae* elicits has historically been attributed to several factors, including hydrogen peroxide and superoxide radicals generated by mycoplasma metabolism, nutritional parasitism, and the host inflammatory responses [13], [14], [15], [16]. Earlier, we proposed that only metabolically active mycoplasmas could introduce toxic factors into respiratory cells resulting in tissue injury [14].

Recently, we described a unique *M. pneumoniae* ADP-ribosylating and vacuolating cytotoxin designated CARDS toxin [17] that shares sequence similarity to the known ADP-ribosylating toxin, *Bordetella pertussis* pertussis toxin S1 subunit, and exhibits an ADP-ribosyltransferase activity in the presence of host cell proteins [12], [17]. Interestingly, rCARDS toxin elicited a distinct vacuolating phenotype in cultured mammalian cell lines, which was not exclusively dependent upon ADP-ribosylating activity [17]. CARDS toxin is the first ADP-ribosyltransferase described that exhibits both ADP-ribosyltransferase and vacuolating activities. rCARDS toxin-induced vacuolation appears particularly relevant to the pathogenic process, as histopathology associated with *M. pneumoniae* infection includes cellular vacuolation, marked deterioration of ciliary movement, and ultimately cell death [15], [18], [19]. Furthermore, baboon tracheal rings exposed to rCARDS toxin exhibited a vacuolated appearance, in addition to a reduction of ciliary movement and loss of cellular integrity of the respiratory epithelium [17]. Importantly, we observed characteristic vacuolation of the bronchial epithelium in rCARDS toxin-treated mice [20].

Toxin-mediated vacuole formation has been extensively examined in *Helicobacter pylori* [21]. Its vacuolating cytotoxin, designated VacA, induces vacuole formation upon acid activation and in the presence of weak bases, like ammonium chloride [22], [23], [24]. Cellular uptake of the acidotropic dye, neutral red, revealed that the intralumenal environment of VacA-induced vacuoles is acidic, and further studies determined the endosomal origin of these vacuoles [25], [26], [27], [28]. The vacuolar membranes of VacA-induced vacuoles were enriched in markers for late endocytic compartments, including lysosome associated membrane protein 1 (LAMP1) and the GTPase, Rab7, but not markers for early endocytic compartments [28]. Rab7 GTPase enrichment onto vacuolar membranes was further linked to its role in vacuole biogenesis [29]. Rab GTPases are key regulators of vesicle traffic in eukaryotic cells and also play an important role in membrane fusion events by recruiting, tethering and docking cellular factors to target membranes. Rab GTPases delineate membrane composition and identity, as they recruit specific effectors to restricted membrane microdomains [30], [31]. Rab5, a protein associated with the plasma membrane and early endosomes, regulates endocytosis at early stages [32]. Rab7 regulates transport from early endosomes to late endosomes [33] or from late endosomes to lysosomes [34] and is associated with vesicular-tubular structures that develop from the perinuclear area to the cellular periphery. Rab9 regulates membrane cycling between late endosomes and the trans-Golgi network (TGN) and is mostly localized to the perinuclear region [35], [36]. While the literature regarding the role of Rab GTPases in membrane trafficking is continually evolving, it has been proposed that VacA-induced vacuolation alters membrane traffic at the late endosomal stage, or inhibits transport, directly or indirectly, between late endosomes and lysosomes [29].

Vacuolation appears to be closely linked to *M. pneumoniae* infection and the presence of CARDS toxin [17], [20]. As CARDS toxin does not share significant sequence similarity with known vacuolating toxins, including VacA, we decided to investigate the formation and composition of vacuoles generated by rCARDS toxin. Using a panel of markers that distinguish vacuole properties, we confirmed the association of a specific, unexpected Rab GTPase with rCARDS toxin-induced vacuoles. Based on these results we propose a novel mechanism of rCARDS toxin-mediated, late endosomal vacuole biogenesis.

## Results

### Characterization of vacuole formation in rCARDS toxin-treated mammalian cells

**Figure 1:**
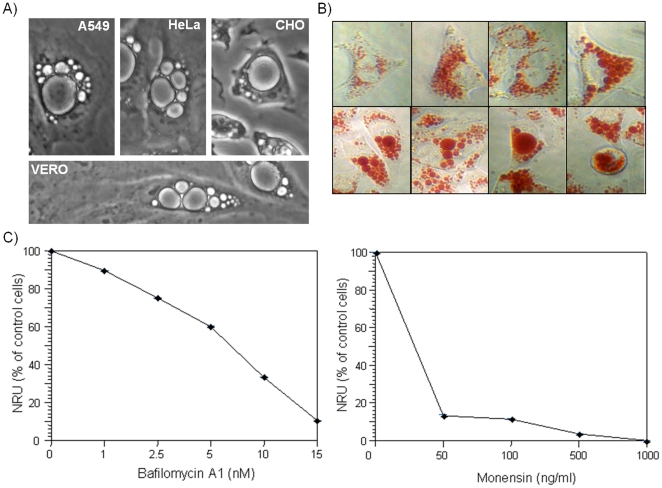
rCARDS toxin-induced vacuolation in mammalian cell lines. (A) A549, HeLa, CHO, and Vero cell monolayers were treated with rCARDS toxin for 24 h at 37°C and images captured Neutral Red Uptake (NRU) by rCARDS toxin-induced HeLa cell vacuoles was observed. Original magnification: ×400. (B) HeLa cells were treated with rCARDS toxin for 24 h at 37°C, and representative images of neutral red-treated cells were compared. Note the diversity of vacuole sizes. (C) HeLa cells pretreated with varying concentrations of bafilomycin A1 or monensin were incubated with rCARDS toxin for 24 h at 37°C. Neutral red in vacuoles was quantified by OD_550_ nm and expressed as percentage of control cells. All values are the mean of two different experiments run in triplicate ± SD (DeltaGraph).

As rCARDS toxin-induced vacuolation of CHO cells was previously demonstrated [17], we analyzed other mammalian cell lines in order to compare the specificity and degree of vacuolization. First, we examined human lung carcinoma epithelial A549 cells, as *M. pneumoniae* is a respiratory pathogen, and rCARDS toxin-induced vacuolation was readily evident (Fig. 1). Other epithelial cell lines examined included kidney (Vero) and cervical (HeLa) cell lines, which exhibited similar vacuolization patterns. rCARDS toxin-mediated vacuolization was dose and time-dependent in all cell lines tested with vacuole formation occurring between 8 to 10 hours at rCARDS toxin concentrations ranging from 5 to 75 µg ml^−1^. Nascent vacuoles first appeared around the perinuclear region, followed by increased numbers and sizes of vacuoles. Within 24 h smaller vacuoles coalesced into larger vacuoles which could occupy the entire cell. As the vacuoles grew in size, they retained more neutral red, indicating their acidic nature and providing a method to quantify rCARDS toxin-induced vacuolation (Fig. 1). When we examined rCARDS toxin-induced vacuole formation in the presence of the V-ATPase inhibitor, bafilomycin A1, decreased neutral red uptake (NRU) correlated with increased concentrations of bafilomycin A1 (Fig. 1). This observation indicated that rCARDS toxin-mediated vacuolation was dependent on V-ATPase enzymes, which are found on the membranes of intracellular organelles, like endosomes and lysosomes. Similarly, decreased NRU was observed in the presence of the ionophore, monensin, reinforcing the acidic intracellular pH of the vacuoles and suggesting an endocytic origin of vacuole formation (Fig. 1).

### rCARDS toxin-induced vacuoles originate along the endocytic pathway

**Figure 2:**
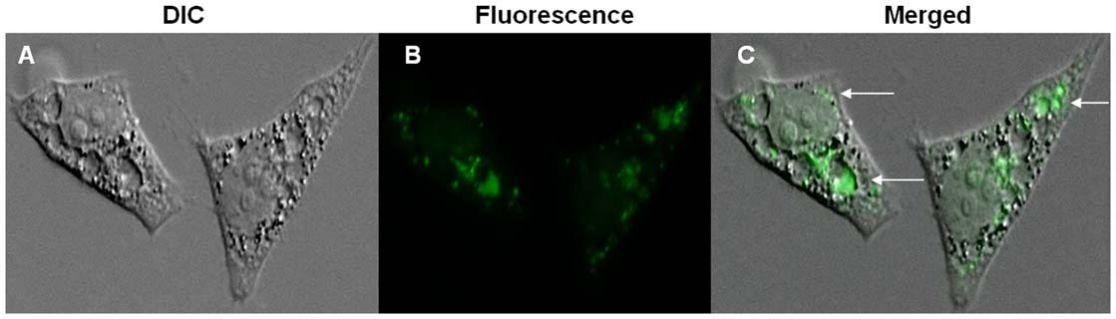
Localization of Lucifer yellow in rCARDS toxin-induced vacuolated HeLa cells. HeLa cells were intoxicated with rCARDS toxin in the presence of 500 µg ml^−1^ Lucifer yellow in MEM medium for 24 h at 37°C. Cells were fixed and visualized by visible light and fluorescence microscopy. (A) DIC; (B) Fluorescence; and (C) Merged image. Lucifer yellow is clearly discernible in a subpopulation of vacuoles (Panel C; see arrows). Original magnification: ×400.

rCARDS toxin-induced vacuoles in HeLa cells accumulate the extracellular fluid-phase marker, Lucifer yellow, indicating that these vacuoles derive from the endocytic pathway (Fig. 2). Fluorescence intensity varied among rCARDS toxin-induced vacuoles, with the majority of smaller vacuoles (98%) exhibiting the most intense fluorescence. In the presence of the cytoskeletal inhibitors, nocodazole and colchicine, toxin-induced vacuolation was markedly reduced at 24 h, as NRU measurements were 4% and 12% of toxin-treated ‘control’ cells, respectively.

### Enrichment of the late endosomal Rab9 GTPase around rCARDS toxin-induced vacuoles

**Figure 3:**
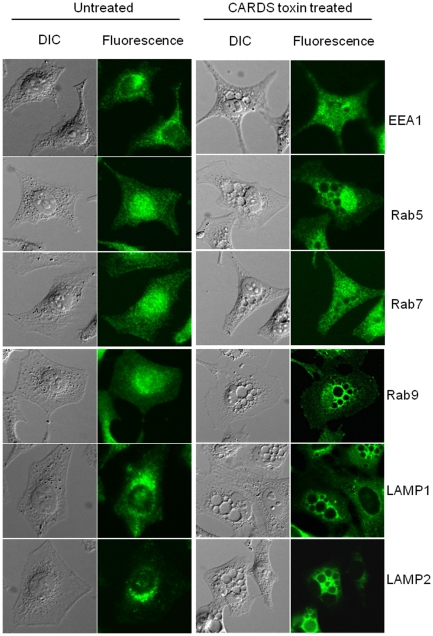
Indirect immunofluoresence analysis of endosomal and lysosomal markers localized to rCARDS toxin-induced vacuoles. Untreated and rCARDS toxin-treated HeLa cells were incubated for 24 h at 37°C and processed as described in Materials and Methods. Representative DIC and fluorescence images of untreated and intoxicated cells are shown. Note that Rab9, LAMP1 and LAMP2 antibodies react with perinuclear region-associated vesicles in untreated cells (left panels) while in intoxicated cells Rab9, LAMP1 and LAMP2 localize and concentrate around specific vacuoles (right panels). Original magnification: ×400.

**Figure 4:**
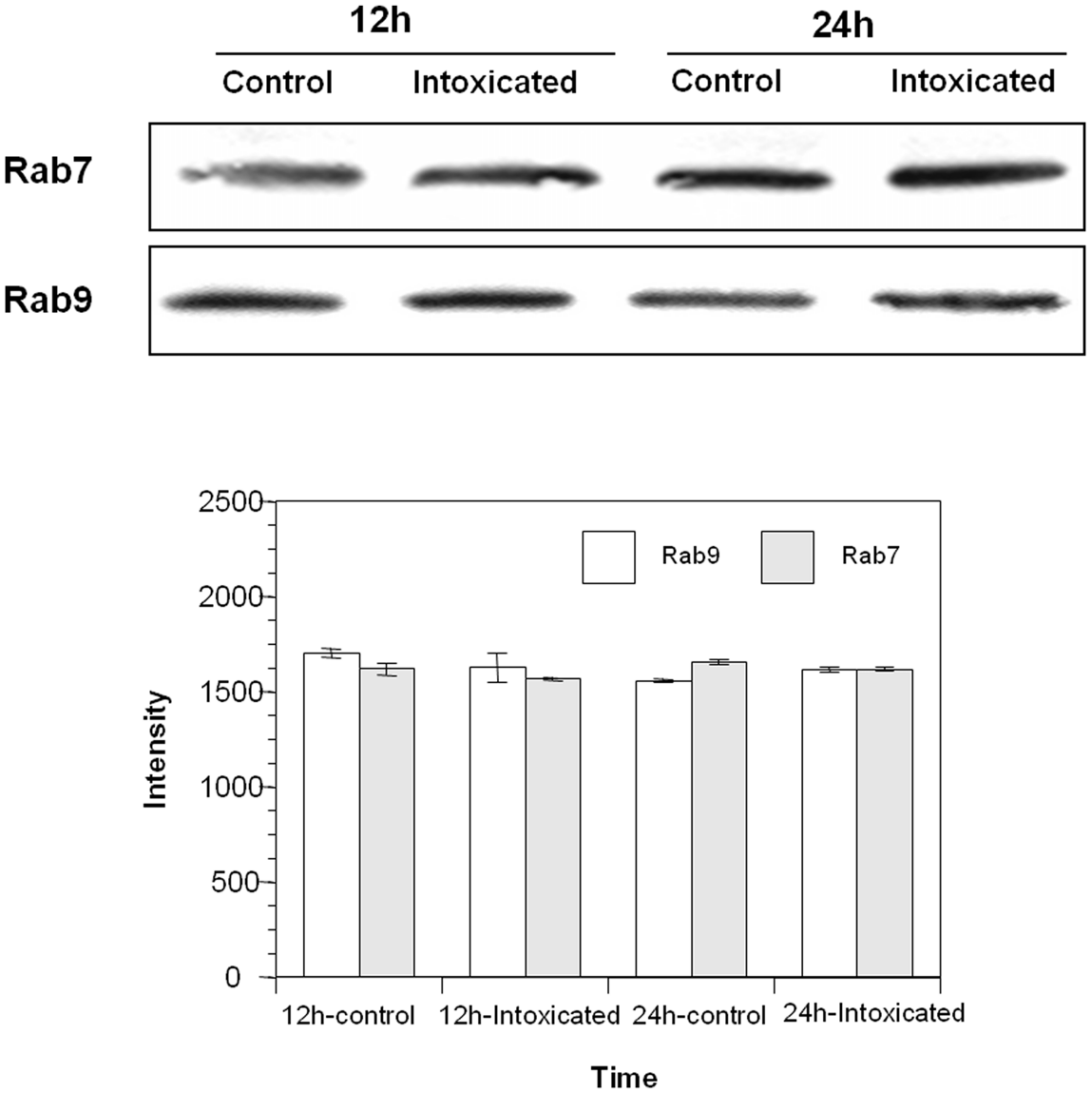
Immunoblot analysis of cellular levels of Rab7 and Rab9 in rCARDS toxin-treated and untreated control HeLa cells. HeLa cells were intoxicated with rCARDS toxin and incubated for 12 or 24 h at 37°C. rCARDS toxin-treated and untreated cell extracts were separated on NuPAGE Bis-Tris 4–12% gels and transferred to nitrocellulose membranes. A) Immunoblot analysis was performed with primary antibodies against Rab9 (mouse) and Rab7 (rabbit) and secondary goat anti-mouse and goat anti-rabbit antibodies conjugated to alkaline phosphatase. B) Quantification of Rab7 and Rab9 band intensities was performed by scanning immunostained bands and analyzing images with KODAK Image software.

As rCARDS toxin-induced vacuoles appear to derive from the endocytic pathway, we examined individual vacuoles for the presence of endosomal and lysosomal markers including Rab5, EEA1, Rab7, Rab9, cathepsin D, LAMP1 and LAMP2 using indirect immunofluorescence. The early endocytic specific markers, Rab5 GTPase and EEA1, were distributed throughout the vacuolated cells in a dispersed pattern with no evidence of selective association with rCARDS toxin-induced vacuoles (Fig. 3). Also, vacuolar membranes did not exhibit discernible selective labeling with the late endosomal GTPase, Rab7. Interestingly, we observed intense enrichment of the late endosomal Rab9 GTPase, as well as LAMP1 and LAMP2, around rCARDS toxin-induced vacuolar membranes (Fig. 3). Since LAMP1 and LAMP2 are known to shuttle between lysosomes, endosomes and the plasma membrane, and since we did not observe an association of rCARDS toxin-induced vacuolar membranes with the lysosomal hydrolase cathepsin D (data not shown), we focused on the late endosomal-specific Rab9 GTPase. We examined whether the enriched Rab9 GTPase around rCARDS toxin-induced vacuoles was due to its overexpression or redistribution by comparing it with another late endosomal marker, Rab7. rCARDS toxin-treated and untreated HeLa cells were analyzed by immunoblot using anti-Rab9 antibodies (Fig. 4). We did not observe increased expression of Rab9 (or Rab7 as negative control) in vacuolated cells at different time points, indicating that the accumulation of Rab9 around rCARDS toxin-induced vacuoles was likely due to redistribution, rather than its increased synthesis during intoxication and vacuole development.

### Distribution and localization of Rab9 at rCARDS toxin-induced vacuolar membranes

**Figure 5:**
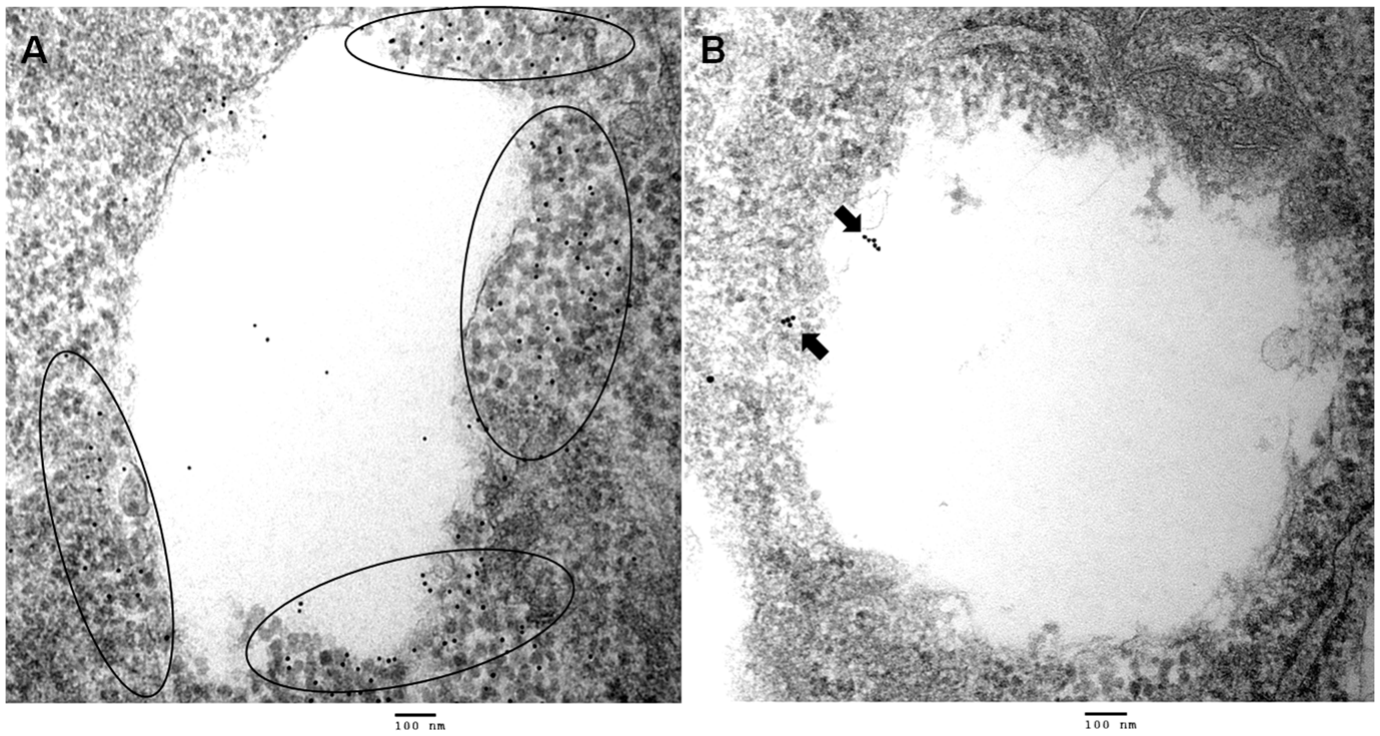
Localization of immunogold-labeled Rab9 to rCARDS toxin-induced vacuoles. CARDS toxin-treated and untreated HeLa cells were incubated for 24 h at 37°C, then labeled with primary antibodies reactive against Rab9 (mouse) or Rab7 (rabbit), followed by goat anti-mouse or goat anti-rabbit IgG gold complex (10 nm), respectively. Representative circles indicate heavy concentration of immunogold labeled Rab9 particles localized to vacuolar membranes in rCARDS toxin-treated cells (A), which are absent in untreated controls. In contrast to Rab9, Rab7 is observed very rarely around the vacuoles in toxin treated vesicles (B). Bar, 100 nm. Original magnification: ×80,000.

Early rCARDS toxin-induced vacuoles were generally delineated by a single bounding membrane, which supported their endocytic origin. Vacuolar fusion events were also observed, accounting for the large size of several vacuoles (5 µm) and leading to their occasional occupation of the entire cell. Consistent with the indirect immunofluoresence results, immunogold-labeled Rab9 was clearly embedded around the margins of CARDS toxin-induced vacuoles (Fig. 5), while immunogold-labeled Rab7 was rarely associated with the vacuolar membranes (Fig. 5). This observation reinforced the specificity of Rab9 binding to toxin-induced vacuoles. Also, gold-conjugated secondary antibodies did not bind to vacuolar membranes, which further supported the unique vacuolar association of Rab9 (data not shown).

### Rab9 is required for rCARDS toxin-induced vacuole formation

**Figure 6:**
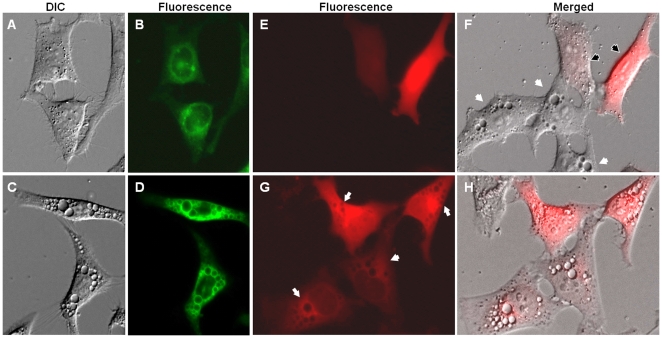
Role of Rab9 in rCARDS toxin-induced vacuoles. HeLa cells were transiently transfected with wild-type pEGFP-Rab9 or pDsRed DN-Rab9 or pDsRed DN-Rab7 and incubated for 24 h at 37°C before addition of rCARDS toxin for an additional 24 h. rCARDS toxin-treated and untreated HeLa cells were analyzed by light and fluorescence microscopy. (A, B) Representative DIC and fluorescence images of untreated HeLa cells transfected with WT pEGFP-Rab9 (C, D) Representative DIC and fluorescence images of rCARDS toxin-treated HeLa cells transfected with WT pEGFP-Rab9. (E, F) Representative fluorescence and fluorescence+DIC merged images of rCARDS toxin-treated HeLa cells transfected with pDsRed DN-Rab9. Note the absence of vacuole formation in DN-Rab9 transfected cells (white arrows and dispersed fluorescence). Also, note distinct vacuoles in non-transfected cells (black arrows and no fluorescence; F). (G, H) Representative fluorescence and fluorescence+DIC merged images of rCARDS toxin-treated HeLa cells transfected with pDsRed DN-Rab7. Note cells transfected with pDsRed DN-Rab7 exhibit distinct vacuoles (H). Original magnification: ×400.

To further confirm the association of rCARDS toxin-induced vacuoles with Rab9, HeLa cells were transiently transfected with either green fluorescent-tagged Rab9 or Rab7 GTPases. We observed the clustering of GFP-Rab9 around vacuolar membranes in intoxicated cells, in contrast to non-intoxicated HeLa cells where GFP-Rab9 was distributed evenly throughout the cytoplasm (Fig. 6). To further delineate the role of Rab9 in vacuole formation, HeLa cells were transiently transfected with mCherry-tagged DN-Rab9. The majority of cells expressing DN-Rab9 lacked visible vacuole development (Fig. 6) supporting the role of Rab9 in rCARDS toxin-mediated vacuole formation. Conversely, HeLa cells expressing DN-Rab7 behaved similarly to toxin-treated, non-transfected cells. In other words, DN-Rab7 transfected cells exhibited rCARDS toxin-induced vacuolation (Fig. 6), which is consistent with the minor or indeterminate role of Rab7 in toxin-induced vacuoles.

## Discussion

*M. pneumoniae* infection is associated with cellular vacuolation, marked deterioration of ciliary movement and ultimately cell death [4], [15]. Interestingly, rCARDS toxin can substitute for live *M. pneumoniae* organisms in eliciting the characteristic airway histopathology in intoxicated mice [20] and tracheal organ cultures [17]. Similarly, rCARDS toxin induces extensive vacuole formation *in vitro* in cultured mammalian cell lines (Fig. 1). These vacuoles initially form around the perinuclear region, fuse, and grow in size until much of the cytoplasm is occupied by a single vacuole or several large vacuoles (Fig. 1). This process would require the progressive addition of membranes, which is consistent with the alteration of membrane trafficking along the endocytic-endosomal pathway. As bacterial toxins have been extremely useful in the study of different aspects of cell biology, the aim of this study was to characterize and elucidate the ontogeny of rCARDS toxin-induced vacuoles.

We observed rCARDS toxin-mediated vacuole formation in multiple cell lines and in a concentration-dependent manner. Similar to VacA, rCARDS toxin-induced vacuoles accumulate neutral red, indicating the acidic nature of the vacuolar lumen [27]. rCARDS toxin distinguishes itself from VacA, as it does not require acid activation or weak bases to induce vacuolation. However, rCARDS toxin-induced vacuolization is prevented by the vacuolar ATPase inhibitor, bafilomycin A1 [37], similar to VacA (Fig. 1). Both bafilomycin A1 and monensin (Fig. 1) are reported to alter intracellular trafficking by preventing early endosome acidification along the endocytic pathway [38], [39] The mechanisms by which both bafilomycin A1 and monensin inhibit rCARDS toxin-mediated vacuole formation are unclear, although similar observations were reported with *Escherichia coli* subtilase cytotoxin [40].

### Endosomal origin of rCARDS toxin-induced vacuoles

The impermeable fluorescent dye, Lucifer yellow, has been used as a marker for fluid phase endocytosis in mammalian cells and *Saccharomyces cerevisiae* [41]. The accumulation of Lucifer yellow within rCARDS toxin-induced vacuoles, especially with the smaller vacuoles (Fig. 2) and the retention of neutral red (Fig. 1) is similar to the *H. pylori* VacA vacuoles [28], revealing their endosomal origin. As cytoskeletal structures are essential for a number of cellular functions, including intracellular transport of endosomes and lysosomes, we analyzed the effect of nocodazole and colchicine on CARDS toxin-induced vacuole formation. These agents prevent polymerization of microtubules and, therefore, depress membrane traffic from early endosomes to late endosomes. As noted earlier, rCARDS toxin-induced vacuolization was markedly reduced by these drugs, further supporting the endocytic nature of these vacuoles [42], [43], [44].

In both untreated and rCARDS toxin-treated cells EEA1 localized to multiple small early endosomal vesicles distributed throughout the cytoplasm with no obvious selective enrichment to toxin-induced vacuoles (Fig. 3). In contrast, we demonstrated that rCARDS toxin-induced vacuolar membranes concentrated Rab9, a small GTPase primarily associated with late endosomes (Fig. 3) [45]. Moreover, we showed an association of LAMP1 and LAMP2 with rCARDS toxin-induced vacuolar membranes. Although LAMP1 and LAMP2 proteins are mainly associated with lysosomes, they also shuttle between endosomes and the plasma membrane [46]. These data favor the possibility that rCARDS toxin-induced vacuoles are derived from late endosomes and that rCARDS toxin may be altering membrane trafficking at this stage. As seen by the neutral red uptake and light microscopy, it is clear that rCARDS toxin vacuoles originate from the perinuclear region and are enriched with Rab9, LAMP1 and LAMP2.

### Rab9 plays an essential role in rCARDS toxin-induced vacuole formation

Interestingly, we did not observe the clustering of another small GTPase associated with late endosomes, Rab7. This finding is in contrast to VacA-induced vacuoles, which contained Rab7 [28], [47]. The functional involvement of Rab9 on *M. pneumoniae* rCARDS toxin vacuole formation was tested by overexpression of DN-Rab9 mutants, which resulted in a significant decrease in rCARDS toxin-induced vacuole development (Fig. 6). These data suggest that the extensive fusion of Rab9-containing late endosomes appears to be crucial for the formation of rCARDS toxin-induced vacuoles. As DN-Rab7 did not interfere with the ability of rCARDS toxin to form vacuoles, in contrast to overexpressed DN-Rab9 (Fig. 6), Rab7 does not appear to play a meaningful role in rCARDS toxin-induced vacuolation. Notably, the late endosomal Rabs, Rab7 and Rab9, have different biological functions and are not always present on the same endosome [45]. Since rCARDS toxin selectively associates with the late endosomal Rab9 GTPase, but not the Rab7 GTPase, we suggest that rCARDS toxin may affect selective subpopulations of late endosomes that are Rab9 positive and Rab7 negative.

In this study we clearly observed the association of Rab9 with rCARDS toxin-induced vacuoles. There is increasing information that viruses and intracellular bacterial pathogens hijack Rab GTPases in order to enter cells through the endocytic pathway, or recruit membranes from the ER or the Golgi apparatus to form replicative intracellular niches [31]. The functions of the Rab GTPases can be modulated by these pathogens to allow their intracellular growth. Numerous studies have shown that selective inclusion or retention of Rab GTPases on vacuolar membranes regulates the biogenesis of phagosomes inhabited by several bacteria. *H. pylori* inhibits phagosome maturation by localizing Rab7 to the bacterium-containing phagosome, while mycobacteria secrete factors that prevent the acquisition of Rab7 by the phagosome, leading to enhanced intracellular survival [30], [48], [49]. *Salmonella typhimurium* acquires multiple Rabs within *Salmonella*-containing vacuoles and *Salmonella*-induced filaments, including Rab7 and Rab9, in order to block fusion with lysosomes and survive intracellularly [50]. Further, it has been found that Rab9 GTPases are key components for replication of several unrelated enveloped viruses, including HIV1, Ebola, Marburg and measles [51]. Since viral assembly initiates at late endosomes for enveloped viruses and viral budding is completed at the plasma membrane, it is hypothesized that these viruses depend on Rab9 to transport viral proteins to the TGN and out to the plasma membrane [51]. As previously mentioned, *M. pneumoniae* is able to establish an intracellular residence, thereby contributing to persistence and chronic airway infections. Whether *M. pneumoniae* utilizes Rab9 in order to achieve this intracellular residence requires further clarification. Alternatively, the endogenous proteins, TGN38 and mannose-6 phosphate-receptor, shuttle between the late endosomes and the TGN, and bacterial toxins, including *Pseudomonas aeruginosa* exotoxin A, have also been shown to utilize the retrograde transport pathway through Rab9 to access the ER [35], [52]. rCARDS toxin may utilize a similar pathway by recruiting Rab9.

With over 70 Rabs identified in the human genome, much remains to be understood regarding the role of these regulatory GTPases in intracellular trafficking, endosomal and phagosomal organelles and microbial pathogenesis. Interestingly, toxins have been helpful in the study of endocytosis and intracellular transport [53]. Future studies with rCARDS toxin may define the molecular mechanisms by which this unique toxin exploits Rab9 vesicles for vacuole formation and delineate how Rab9 connects to various components of the transport machinery, such as cargo adaptors, molecular motors, vesicle fusion protein and other Rab GTPases.

As mentioned earlier, hallmarks of *M. pneumoniae* infection are characteristic and programmed injury to respiratory cells with accompanying histopathology and inflammatory responses. As it became evident that rCARDS toxin was sufficient in replacing live *M. pneumoniae* in the production of vacuolization, we focused on understanding toxin-mediated vacuolating pathways. Here, we demonstrate that rCARDS toxin-induced vacuoles originate from late endosomal compartments and recruit the small GTP-binding protein, Rab9, which distinguishes CARDS toxin from VacA. Further investigation into the origin and mechanisms of CARDS toxin-induced vacuolation may clarify the role of *M. pneumoniae* and its ADP-ribosylating and vacuolating CARDS toxin in infection and disease progression.

## Materials and Methods

### Chemical reagents and antibodies

Neutral red, bafilomycin A1, colchicine, nocodazole, and monensin were purchased from Sigma and Lucifer yellow from Molecular Probes (Invitrogen). Antibodies reactive against EEA1, Rab7, cathepsin D, LAMP1, and LAMP2 were obtained from Abcam. Rab 5 antibody was purchased from Cell Signaling Technology and Rab 9 antibody from Novus Biologicals. Alexa Fluor 488 goat anti-mouse and goat anti-rabbit affinity-purified antibodies were purchased from Invitrogen.

### Expression and purification of rCARDS toxin

rCARDS toxin was expressed in BL21 DE3 (LpxM) [54], purified using nickel column chromatography (Qiagen), and desalted using PD10 desalting columns (GE Healthcare) as previously described by us [17]. Similarly expressed and purified *M. pneumoniae* recombinant EF-Tu and recombinant pyruvate dehydrogenase ß subunit proteins were used as negative controls [17].

### Mammalian cell growth conditions

HeLa (CCL-2), Vero (CCL-81), CHO-K1 (CCL-61), and A549 (CCL-185) cells were obtained from the American Type Culture Collection (ATCC). HeLa and Vero cells were passaged in Minimal Essential Medium (MEM) (ATCC) with 10% fetal bovine serum (FBS) (Atlas Biologicals). CHO-K1 (CCL-61) and A549 (CCL-185) cells were passaged in F-12K medium (ATCC) with 10% FBS. All cell lines were maintained at 37°C in 5% CO_2_ in 75 cm^2^ flasks (Corning).

### rCARDS toxin-induced vacuolation of mammalian cell lines

Mammalian cell lines were grown in T25 cm^2^ flasks (Corning) and incubated in the presence or absence of rCARDS toxin at varying concentrations and time intervals at 37°C. Cell vacuolation was visualized using inverted light (Olympus CK 40) or differential interference contrast (DIC) microscopy (Carl-Zeiss Cell Observer).

### Quantification of rCARDS toxin-induced vacuolation by NRU

The NRU assay was performed as described with slight modification [55]. Briefly, HeLa cells were seeded in 96-well plates at a concentration of 4×10^4^ ml^−1^ for 24 h, before addition of rCARDS toxin at varying concentrations (5 µg to 75 µg ml^−1^) for different time points. For specific experiments, HeLa cells were pre-incubated with nocodazole (250 ng ml^−1^) and colchicine (10 µM) for 1 h before addition of 50 µg ml^−1^ rCARDS toxin for 24 h at 37°C. Then, MEM culture medium was aspirated from HeLa cells, and 100 µl of neutral red solution (40 mg ml^−1^ neutral red prepared in phosphate buffered saline [PBS pH 7.4], filter sterilized and diluted 1∶100 in MEM medium) were added to each well. Incubation was continued for 2 h at 37°C; cells were washed with PBS; neutral red was extracted with 150 µl of destain solution (50% ethanol, 49% distilled water, and 1% glacial acetic acid); and OD_550_ nm was determined using a microtiter plate reader spectrophotometer (Opsys MR, Dynex Technologies Inc.). NRU was also measured in the presence and absence of monensin and bafilomycin A1.

### Cytochemical staining of rCARDS toxin-induced vacuolation

Movement and localization of Lucifer yellow carbohydrazide into vacuoles were assessed by incubating HeLa cells in the presence of 75 µg ml^−1^ rCARDS toxin and 500 µg ml^−1^ Lucifer yellow in MEM medium for 24 h at 37°C. Cells were washed with PBS, fixed in 2% paraformaldehyde (methanol free, Electron Microscopy Sciences) in PBS, and visualized with DIC and 488 filter sets using a Carl Zeiss Cell Observer Z.1 microscope operated by a multichannel acquisition system (AXIO Vision ver.4.7.2). Small and large vacuoles enriched with Lucifer yellow were counted (>500 vacuoles counted) to analyze the percentage of vacuoles associated with Lucifer yellow.

### Immunofluorescence analysis of endosomal membrane markers localized to rCARDS toxin-induced vacuoles

HeLa cells were seeded in 4-chamber glass slides (Lab-Tek II Chambered #1.5 Borosilicate Coverglass, Fisher) at a concentration of 5×10^4^ cells/chamber 24 h before intoxication. Then, cells were incubated in the presence or absence of 75 µg ml^−1^ rCARDS toxin for 24 h at 37°C. Cells were washed once with PBS, fixed in 2% paraformaldehyde (methanol free) in PBS for 15 min at room temperature (RT), washed with PBS; and permeabilized in 0.2% Triton X-100 in PBS for 5 min. Cells were again washed in PBS with 1% normal goat serum (NGS, GIBCO) and incubated with primary antibodies diluted 1∶100 (EEA1, Rab 5, Rab 7, Rab 9, Cathepsin D, LAMP1, and LAMP2) in PBS with 0.2% NGS for 1 h. Cells were washed with PBS containing 0.2% NGS and incubated with secondary antibodies diluted 1∶500 (Alexa Fluor 488 goat anti-mouse or goat anti-rabbit) in PBS with 0.2% NGS for 1 h at RT. Cells were washed again with PBS and visualized with DIC and 488 filter sets using a Carl-Zeiss Cell Observer Z.1 microscope.

### Comparative immunoblot analysis of endosomal membrane markers in rCARDS toxin-treated and untreated HeLa cells

HeLa cells grown in T25 cm^2^ flasks were incubated in the presence or absence of 75 µg ml^−1^ rCARDS toxin for 24 h at 37°C. After visual detection of vacuolation by light microscopy, HeLa cells were washed and scraped into PBS, and protein concentrations were determined by bicinchoninic acid (BCA) quantitation (Pierce). Intoxicated and non-intoxicated HeLa cells were boiled in SDS-PAGE loading buffer, and total cell protein (2 µg/well) was separated by gel electrophoresis on 4–12% NuPAGE gels and transferred to nitrocellulose membranes. Membranes were blocked in 5% blotto in Tris-Buffered Saline-Tween-20 (TBST) for 1 h at RT. Primary antibodies were diluted 1∶100 in 3% blotto in TBST and incubated with membranes for 1 h. Secondary goat anti-rabbit (Zymed) and goat anti-mouse antibodies (Zymed) were diluted in TBST at 1∶3000.

### Immunoelectron microscopic analysis of rCARDS toxin-treated HeLa cells

HeLa cells grown in T25 cm^2^ flasks were intoxicated with 75 µg ml^−1^ rCARDS toxin or buffer alone and incubated overnight at 37°C. HeLa cells were washed with PBS and fixed with 1% glutaraldehyde/4% formaldehyde for 2 h at RT. Then, cells were scraped into fixative and sections prepared and mounted on Formvar-coated nickel grids. Individual grids were blocked with 100 mM Tris-HCl containing 1% BSA and 1% NGS and incubated with antibodies reactive against Rab7 (rabbit polyclonal; 1∶10 dilution) or Rab9 (mouse monoclonal; 1∶10 dilution) in PBS containing 1% BSA. After three washes in PBS with 1% BSA, grids were incubated with secondary goat anti-rabbit 20 nm gold particles (Rab7) and goat anti-mouse 10 nm gold particles (Rab9) antibodies diluted 1∶10 in PBS with 1% BSA. Individual grids were analyzed with a JEOL 1230 transmission electron microscope at an 80-kV accelerating voltage after staining with 7% uranyl acetate followed by Reynold's lead citrate.

### Transient transfection of wild-type (WT) and dominant-negative (DN) Rab7 and Rab9 in HeLa cells

HeLa cells were seeded in 4-chamber glass slides at a concentration of 5×10^4^ cells/chamber for 24 h at 37°C prior to transfection. Cells were either transfected with pEGFP-rab7 WT (Addgene plasmid 12605), pEGFP-rab9 WT (Addgene plasmid 12663), DsRed-rab9 DN (Addgene plasmid 12676), or DsRed-rab7 DN (Addgene plasmid 12662) [56] using FuGENE 6 transfection reagent (Roche) according to manufacturer's instructions. Twenty-four h after transfection, cells were intoxicated with 75 µg ml^−1^ rCARDS toxin, incubated for another 24 h, and washed with PBS. Cells were visualized with DIC and 488 or 633 filter sets using a Carl Zeiss Cell Observer Z.1 microscope.
